# *In silico* molecular target prediction unveils mebendazole as a potent MAPK14 inhibitor

**DOI:** 10.1002/1878-0261.12810

**Published:** 2020-10-18

**Authors:** Jeremy Ariey‐Bonnet, Kendall Carrasco, Marion Le Grand, Laurent Hoffer, Stéphane Betzi, Mikael Feracci, Philipp Tsvetkov, Francois Devred, Yves Collette, Xavier Morelli, Pedro Ballester, Eddy Pasquier

**Affiliations:** ^1^ Centre National de la Recherche Scientifique (CNRS) Institut National de la Santé et de la Recherche Médicale (INSERM) Institut Paoli Calmettes Centre de Recherche en Cancérologie de Marseille (CRCM) Aix Marseille Université France; ^2^ CNRS UMR 7051 INP Inst Neurophysiopathol Fac Pharm Aix Marseille Université France; ^3^ Present address: CNRS UMR 7257 Architecture et Fonction des Macromolécules Biologiques Aix‐Marseille Université Marseille France

**Keywords:** cancer, drug target prediction, glioblastoma, MAPK14, mebendazole, polypharmacology

## Abstract

The concept of polypharmacology involves the interaction of drug molecules with multiple molecular targets. It provides a unique opportunity for the repurposing of already‐approved drugs to target key factors involved in human diseases. Herein, we used an *in silico* target prediction algorithm to investigate the mechanism of action of mebendazole, an antihelminthic drug, currently repurposed in the treatment of brain tumors. First, we confirmed that mebendazole decreased the viability of glioblastoma cells *in vitro* (IC_50_ values ranging from 288 nm to 2.1 µm). Our *in silico* approach unveiled 21 putative molecular targets for mebendazole, including 12 proteins significantly upregulated at the gene level in glioblastoma as compared to normal brain tissue (fold change > 1.5; *P* < 0.0001). Validation experiments were performed on three major kinases involved in cancer biology: ABL1, MAPK1/ERK2, and MAPK14/p38α. Mebendazole could inhibit the activity of these kinases *in vitro* in a dose‐dependent manner, with a high potency against MAPK14 (IC_50_ = 104 ± 46 nm). Its direct binding to MAPK14 was further validated *in vitro*, and inhibition of MAPK14 kinase activity was confirmed in live glioblastoma cells. Consistent with biophysical data, molecular modeling suggested that mebendazole was able to bind to the catalytic site of MAPK14. Finally, gene silencing demonstrated that MAPK14 is involved in glioblastoma tumor spheroid growth and response to mebendazole treatment. This study thus highlighted the role of MAPK14 in the anticancer mechanism of action of mebendazole and provides further rationale for the pharmacological targeting of MAPK14 in brain tumors. It also opens new avenues for the development of novel MAPK14/p38α inhibitors to treat human diseases.

## Graphical abstract

Drug polypharmacology was explored *in silico* to identify novel molecular targets of antihelminthic drug mebendazole, currently repurposed for the treatment of brain cancers. By combining transcriptomic analysis, molecular docking, and functional assays (biophysical, biochemical, and cellular), we validated MAPK14 as a critical target of mebendazole and a key player in glioblastoma progression.

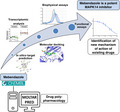

**Abbreviations**
BRET: bioluminescence resonance energy transfer
GBM: glioblastoma
GTeX: Genotype‐Tissue Expression
IC_50_: half‐maximal inhibitory concentration
ITC: isothermal titration calorimetry
MBZ: mebendazole
nanoDSF: nanoscale differential scanning fluorimetry
qRT‐PCR: quantitative real‐time polymerase chain reaction
RT: room temperature
siRNA: small interfering RNA
TCGA: The Cancer Genome Atlas
TSA: thermal shift assay

## 1 Introduction

The concept of polypharmacology, which involves the interaction of drug molecules with multiple targets, has emerged in recent years as a new paradigm in drug development [1, 2]. Polypharmacology can either limit or expand the medical indications of pharmacological agents. On the one hand, unintended drug–target interactions can cause toxic side effects, which can restrict and even prevent clinical use of a given drug. On the other hand, multitargeting activity can open new therapeutic avenues in cancer research. Indeed, it is generally thought that complex diseases such as cancer arise from numerous alterations. A drug that ‘hits’ multiple sensitive nodes belonging to a network of interacting targets thus offers the potential for higher efficacy that might require lower dosage and therefore induce fewer toxic side effects. In this context, using already‐approved drugs—a concept called drug repurposing—could be of interest. Indeed, deciphering the polypharmacological profile of already‐approved drugs has the potential to fast‐track the discovery of new efficient drugs as their toxic profiles are already known, while highlighting new drug–target interactions. An early estimation of the degree of drug polypharmacology using seven different databases identified a total of 4767 unique interactions between 802 drugs and 480 targets, suggesting an average of 6 molecular targets per approved drug [3]. Recently, Peón *et al*. [4] updated this estimation to an average of 11.5 molecular targets per drug in cells obtained from a more complete dataset comprising 8535 drug–target associations and 1427 targets. This high degree of polypharmacology thus provides a unique opportunity for the repurposing of already‐approved drugs to target key factors involved in human diseases.

One of the main limitations of drug development is its elevated cost. Thus, the rapid identification of novel therapeutic targets for already‐approved drugs provides a route to reduce development cost by expanding their indications to human conditions in which those targets have been validated [5, 6]. Here, we applied a multipronged approach to unveil and validate new molecular targets for a well‐known repurposed drug called mebendazole (MBZ). This antihelminthic agent, which belongs to the benzimidazole class, has been shown to display potent anticancer properties in various models of human cancers [7, 8, 9, 10, 11, 12] and thus appears as a promising candidate for drug repurposing in oncology. Although the discovery of its therapeutic potential in brain tumors was fortuitous [11], it already resulted in three ongoing clinical trials in high‐grade gliomas in both adult and pediatric patients (NCT01729260, NCT02644291, and NCT01837862). Several mechanisms of action have been proposed to explain the anticancer properties of MBZ. These include tumor angiogenesis inhibition [12, 13], targeting of critical pathways involved in cancer such as Hedgehog signaling [14], and stimulation of anticancer immune response [15, 16]. Most of these effects have been linked to the ability of MBZ to induce microtubule depolymerization in cancer cells [8, 11, 14, 17, 18]. However, the affinity of MBZ for human tubulin is lower than that of helminthic tubulin [19]. Furthermore, the toxic side effects of MBZ are different and significantly milder than those of conventional microtubule‐targeting chemotherapy agents, taxanes and *Vinca* alkaloids. This strongly suggests that additional, yet unknown mechanisms may be involved in the anticancer activity of MBZ, warranting further investigation.

Herein, we used *in silico* drug target prediction to identify novel putative molecular targets of MBZ in glioblastoma (GBM) cells. Through experimental validation of the predicted targets, we discovered that MBZ binds to MAPK14/p38α and inhibits its kinase activity *in vitro* and *in cellulo*. In accordance with biophysical characterization, molecular modeling studies predicted that MBZ was able to bind the catalytic site of MAPK14. Finally, gene silencing by RNA interference confirmed that MAPK14 plays a key role in the cytotoxic activity of MBZ against GBM cells and represents a promising therapeutic target in GBM.

## 2 Materials and methods

### 2.1 Cell culture

U87, U87vIII, T98G, and U251 are glioblastoma cell lines. They were grown in Dulbecco's modified Eagle's medium (Thermo Fisher Scientific, Villebon‐sur‐Yvette, France) containing 10% fetal bovine serum and 1% pyruvate and 1% penicillin–streptomycin. They were routinely maintained in culture on 0.1% gelatin‐coated flasks at 37 °C and 5% CO_2_. Both cell lines were regularly screened and are free from mycoplasma contamination.

### 2.2 Cell viability assay

Cell viability assays were performed as previously described [20]. Briefly, cells were seeded at 4500 cells/well in 96‐well plates. After 24 h, cells were treated with a range of concentrations of benzimidazole agents, and after 72‐h drug incubation, metabolic activity was detected by addition of Alamar Blue and spectrophotometric analysis using a PHERAstar plate reader (BMG LABTECH, Champigny‐sur‐Marne, France). Cell viability was determined and expressed as a percentage of untreated control cells. The determination of IC_50_ values was performed by using the following equation: *Y* = 100/(1 + ((*X*/IC_50_)^Hillslope)).

### 2.3 Gene expression analysis on patient samples

Gene expression analysis was conducted using the R2 microarray analysis and visualization platform (http://r2.amc.nl). RNA‐Seq data were extracted from two independent cohorts providing open access to data acquired from various forms of cancer: the Cancer Genome Atlas (TCGA) database, and from normal tissues: the Genotype‐Tissue Expression (GTeX) database. GBM TCGA dataset was used and partitioned in five subtypes according to the data available: classical (*n* = 17), mesenchymal (*n* = 27), neural (*n* = 17), proneural (*n* = 24), and not determined (*n* = 455). We used GTeX normal brain tissue data from the following subgroups: caudate (*n* = 246), cortex (*n* = 255), frontal cortex (*n* = 209), nucleus accumbens (*n* = 246), and putamen (*n* = 205). Median values were recorded using log2 transformation gene expression. Statistical analyses using ANOVA were performed to compare GBM subtype gene expression to normal brain tissue gene expression. Box plots representing average values were generated using graphpad prism 8.4.1 (Graphpad Software Inc., La Jolla, CA, USA).

### 2.4 Kinase assay

MAPK14, ERK2, and ABL1 kinase assay were purchased from Promega (Charbonnieres‐les‐bains, France). Enzyme, substrate, ATP, and inhibitors were diluted in Kinase Buffer as per the manufacturer's instructions. Kinase reaction was performed in 384‐well plate in a final volume of 5 μL. Reaction was initiated using 1 μL of inhibitor for each concentration (1% DMSO), 1 μL of enzyme, and 3 μL of substrate/ATP mix (60 min, RT). Five microlitre of ADP‐GloTM reagent was used to stop kinase reaction by ATP depletion (40 min, RT). Then, ADP formed by kinase reaction was detected by adding 10 μL of Kinase Detection Reagent (30 min, RT). Luminescence was recorded using a PHERAstar plate reader. MAPK14, ERK2, and ABL1 kinases were used at optimized concentrations of 4, 3, and 1 ng/well, respectively. For the three proteins, ATP was used at 5 μm and DTT at 50 μm. The substrates of MAPK14, ABL1, and ERK2 were used at 0.2, 0.2, and 0.1 μg·μL^−1^.

### 2.5 NanoBRET target engagement assay

U87 cells were transfected with MAPK14‐NanoLuc fusion vector DNA (Promega) using Lipofectamine™ RNAiMAX (Life Technologies, Villebon‐sur‐Yvette, France) and following the manufacturer's instructions. 17 000 cells/well were dispensed into a 96‐well NBS plates and prepared with 0.1 μm of NanoBRET™ Tracer K‐4 reagent. Cells were treated with MBZ at concentrations ranging from 0.05 to 10 μm and incubated at 37 °C, 5% CO_2_ for 2 h. A separate set of samples without tracer was prepared for background correction. Plate was equilibrated at RT during 15 min. Complete substrate plus inhibitor solution in assay medium (Opti‐MEMRI reduced serum medium, no phenol red) was prepared just before measuring BRET signal. Fifty microlitre of 3X complete substrate plus inhibitor solution was added to each well of the 96‐well plate and incubated for 2–3 min at RT. Donor emission wavelength (460 nm) and acceptor emission wavelength (610 nm) were measured using a PHERAstar plate reader.

### 2.6 Protein expression and purification

For MAPK14 expression, we used the pET28a vector kindly provided by Qi and Huang of the National Institute of Biological Sciences in China [21]. The expression and purification of wild‐type MAPK14 were carried out as previously reported [22]. For the isothermal titration calorimetry (ITC) and the thermal shift assay (TSA), the His tag was conserved, and the protein was concentrated to 17 mg·mL^−1^ in 25 mm Tris/HCL pH 7.4, 150 mm NaCl, 5% glycerol, 10 mm MgCl_2_, and 5 mm DTT. The samples were immediately flash‐frozen in liquid nitrogen and stored at −80 °C. A pET28a vector was also used to perform the expression of the kinase domain of human c‐ABL (ABL1). The protein was expressed in *Escherichia coli* strain BL21 (DE3) STAR in TB media with 50 µg·mL^−1^ kanamycin and 34 µg·mL^−1^ chloramphenicol at 17 °C overnight after induction with 0.2 mm of IPTG. The bacteria were disrupted by sonication on ice for 3 min in lysis buffer (50 mm Tris/HCl pH 8, 500 mm NaCl, 5% glycerol, 10 mm imidazole, and 0.1% BRIJ35) with EDTA‐free protease inhibitor cocktail (Sigma Aldrich Chimie, Saint Quentin Fallavier, France). The protein from the soluble fraction was loaded onto a HisTrap Ni‐NTA column, washed with 5 column volumes of lysis buffer containing 10 mm Imidazole and 5 volumes of 40 mm imidazole buffer, and eluted with 5 volumes of a gradient buffer from 40 to 500 mm imidazole. The resulting protein was concentrated and purified by size‐exclusion chromatography using Superdex 75 (GE Healthcare, Velizy, France) with 10 mm Tris pH 8.0, 50 mm NaCl, and 1 mm DTT.

### 2.7 Thermal shift assay

Thermal shift assay experiments were performed in triplicate in 384‐well PCR plates (Bio‐Rad, Marnes‐la‐coquette, France). The reagents (compound, protein, and fluorophore) were dispensed using an Echo 550 acoustic dispenser (Labcyte, San Jose, CA, USA): 100 nL of compound (from a 100% DMSO stock at 10 mm) for a final concentration of 50 μm (0.5% final DMSO); 200 nL of Thermal Shift Dye (Thermo Fisher Scientific) diluted to a final concentration of 0.1%; and 300 nL of MAPK14 (42 μm stock) for a final concentration of 5 μm. The final assay volume was completed to 19.5 μL with assay buffer (10 mm HEPES, pH 7.5, 500 mm NaCl) using a Multidrop Combi (Thermo Fisher Scientific). For TSA experiments on ABL1, the kinase domain of human c‐ABL (ABL1) was mixed at a final concentration of 4 µm with the inhibitor (200 µm final concentration/2% DMSO) in a final volume of 20 µL of the assay buffer (25 mm HEPES pH 7.5/150 mm NaCl/1 mm DTT/1% glycerol). For both assays, the Thermal Shift Dye was added at the end diluted to a final concentration of 0.1%. The plates were sealed with optical film (AMPLIseal, Greiner, Les Ulis, France) and centrifuged at 300 ***g*** for 1min at 4 °C. The thermal melting experiments were carried using a CFX384 RT‐PCR (Bio‐Rad). The plates were first equilibrated at 25 °C for 1min and then heated using a 0.5 °C steps ramp from 25 °C to 95 °C using 25‐s equilibration. Raw fluorescence was measured, and the melting temperatures (Tm) were calculated using cfx manager 3.1 (Bio‐Rad).

### 2.8 Isothermal titration calorimetry

Isothermal titration calorimetry was used to determine the thermodynamics parameters of the binding between MAPK14 and the selected compounds. Titrations were carried out on a MicroCal iTC200 microcalorimeter (GE Healthcare). Each experiment was designed as normal (protein in the syringe and ligand in the cell) or reverse (protein in the syringe and ligand in the cell) titrations experiments using 13 to 17 injections at 15 °C or 25 °C. Raw data were scaled after setting the titration–saturation–heat value to zero. Integrated raw ITC data were fitted to a one‐site nonlinear least‐squares‐fit model using the MicroCal Origin plug‐in as implemented in origin 9.1 (Origin Lab, Northampton, MA, USA). Finally, Δ*G* and *T*Δ*S* values were calculated from the fitted Δ*H* and KD values using the equations Δ*G* = −RTlnKD and Δ*G* = Δ*H* − *T*Δ*S*. Each experiment was performed as triplicates, and data are presented as the mean ± SD.

### 2.9 Nanoscale differential scanning fluorimetry (nanoDSF)

The thermostability of MAPK14 was measured in the presence of different concentrations of MBZ in 50 mm HEPES buffer in the presence of 200 mm NaCl and 2% DMSO at pH 7.5 using a label‐free fluorimetric analysis with a Prometheus NT.Plex instrument (NanoTemper Technologies, München, Germany), as described previously [23]. NanoDSF‐grade capillaries were filled with a 10 µm solution of interest. The concentration of MBZ varied from 0.78 to 200 µm, while MAPK14 concentration was fixed at 5 µm. Capillaries were loaded into the Prometheus NT.Plex and heated from 25 °C to 70 °C with a 1 K·min^−1^ heating rate at low detector sensitivity with an excitation power of 10%. Unfolding transition points (Tm) were determined from the first derivative of the changes in ratio between the emission wavelengths of tryptophan fluorescence at 330 and 350 nm, which were automatically identified by the prometheus nt.plex control software (NanoTemper Technologies).

### 2.10 Molecular modeling

The PDB files (1A9U and 3FLY) were prepared using moe version 2016 (http://chemcomp.com). The binding site was defined as all residues with at least one atom within 10 Å radius from the crucial M109 known hot spot. The studied compounds, including cocrystallized ligands and MBZ‐like inhibitors, were also prepared using moe in order to generate random 3D conformers and compute required partial charges. PLANTS was used as the docking engine with its ‘Chemplp’ scoring function to generate and evaluate the poses [24]. Its docking algorithm is based on a class of stochastic optimization algorithms called ant colony optimization. This kind of algorithm, which mimics the behavior of ants finding the shortest path between food and their nest, can be used to efficiently sample the conformational space for docking purpose. Neither geometric nor pharmacophore constraints were added in simulations. Redocking experiments of reference ligands (SB203580 and pyrido‐pyrimidin inhibitor) into their respective structures (1A9U and 3FLY) were beforehand performed to validate the docking settings and protocol. This control study is used to determine the best docking parameters for the target [25]. Both ligands were successfully redocked into their binding site with a RMSD value of less than 1 Å between crystallized conformation and predicted pose. All poses from docking simulations were subjected to visual analysis using pymol (http://pymol.org), and the binding mode of interest was selected accordingly. The latter should (a) contain polar interactions with crucial hot spot M109, (b) highlight shared binding mode between all active MBZ analogs, (c) be compatible with known SAR data, and (d) be reasonable in terms of docking score and explicit interactions with the binding site. The most promising poses were subjected to postprocessing with the commercial seesar software (http://biosolveit.de). Briefly, this computer‐aided design tool, which was developed for modelers and chemists, relies on the HYDE method [26] to evaluate affinity contributions to the binding for each ligand atom. seesar can then be used to (a) optimize docking poses within the binding site, (b) identify optimal and suboptimal moieties from ligands, (c) analyze SAR data from 3D complex point of view, and (d) design and evaluate new analog compounds in order to optimize the series. In this work, seesar was initially used to identify potential issues in prioritized poses, mainly desolvation penalties, low‐quality hydrogen bonds, and suboptimal torsions. Ultimately, seesar was used to estimate the affinity of each compound from the MBZ series from their respective postprocessed binding mode. Additional PDB files were used for the seesar‐based reevaluation of the predicted binding mode of MBZ within the binding sites of other considered kinases from Table 2 (4ZZN for MAPK1/ERK2 and 1M52 for ABL1). Both X‐ray structures include wild‐type proteins and contain small organic ligands with similar binding mode with respect to the reference 3FLY structure.

### 2.11 Functional validation of MAPK14

DsRed‐expressing U87 cells were seeded in T25 flask cell culture and transfected with 1 mL of Opti‐MEM medium containing 1% of Lipofectamine RNAiMAX and 5 nm of siRNA. Three different siRNA sequences targeting MAPK14 were used (Silencer^®^ Select s3585, s3586, and s3587; Thermo Fisher Scientific) as well as a nontargeting negative control siRNA with no significant sequence similarity to mouse, rat, or human gene sequences (Silencer^®^ Select AM4635). Two days later, cells were seeded in 96‐well U bottom and low‐binding plates in DMEM containing methylcellulose at 0.6 g·L^−1^ and spheroids were treated with increasing concentrations of MBZ 24 h after seeding. Spheroid viability was then evaluated daily for 1 week by measuring the dsRed fluorescence ratio (575 nm: excitation wavelength/620 nm: emission wavelength) with a PHERAstar plate reader.

To evaluate the level of gene knockdown, cells were harvested 48 h after transfection and total RNA was extracted using RNeasy Kit (Qiagen, Les Ulis, France), following the manufacturer's instructions. Reverse transcription was performed with OneScript^®^ cDNA Synthesis Kit (Abm, Applied Biological Materials, Vancouver, Canada), and qRT‐PCR was performed using SsoAdvanced Universal SYBR^®^ Green Supermix (Bio‐Rad) and a CFX96™ Real‐Time System Device (Bio‐Rad). Gene expression levels were determined using the ΔΔ*C*_t_ method, normalized to the *YWHAZ* control gene. The following predesigned KiCqStart SYBR^®^ Green primers (Merck, Fontenay‐sous‐bois, France) were used: *MAPK14* (forward: 5′‐AGATTCTGGATTTTGGACTG; reverse: 5′‐CCACTGACCAAATATCAACTG) and *YWHAZ* (forward: 5′‐AACTTGACATTGTGGACATC; reverse: 5′‐AAAACTATTTGTGGGACAGC).

## 3 Results

### 3.1 Mebendazole exerts potent antiproliferative effects against GBM cell lines *in vitro*

**Fig. 1:**
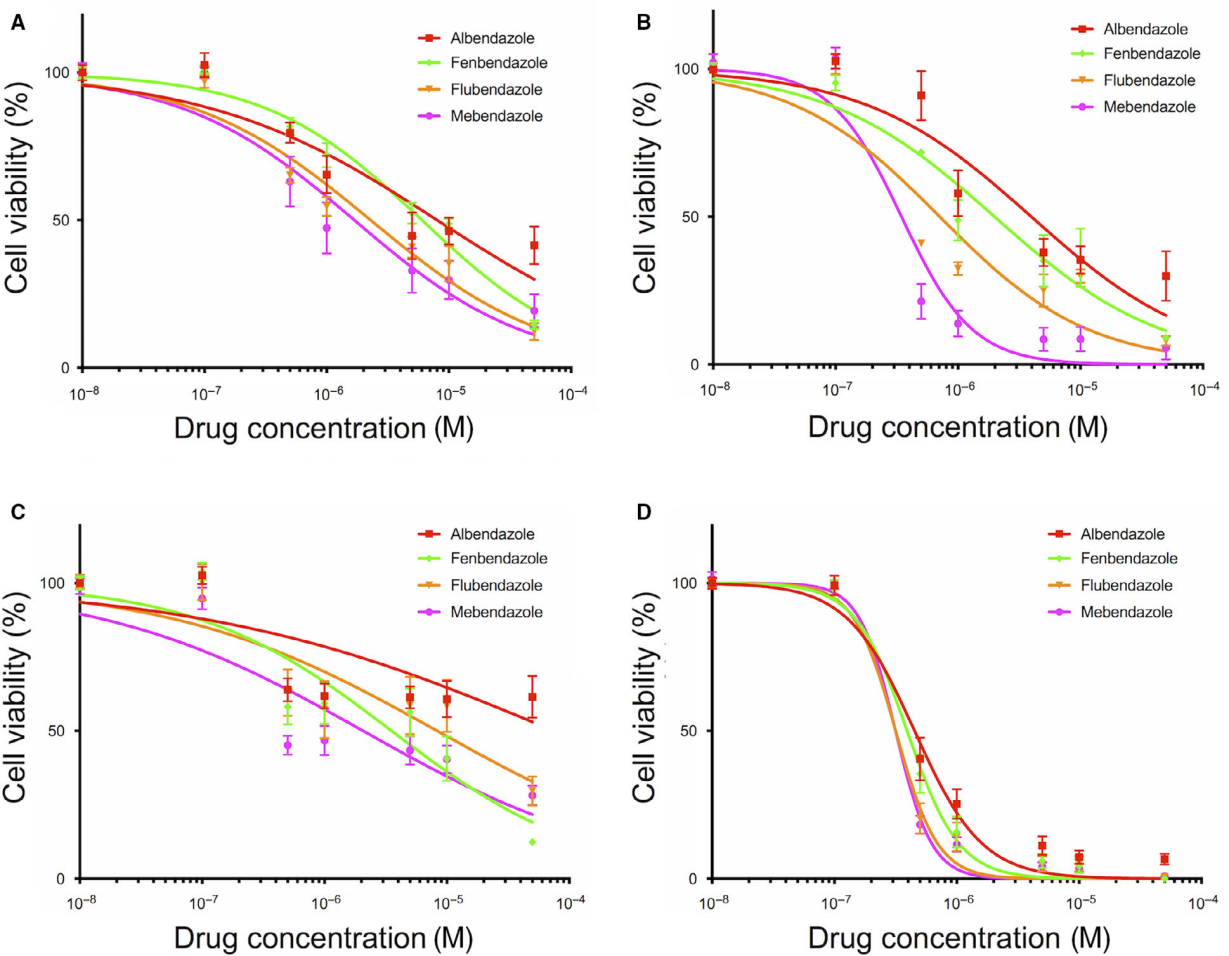
Impact of benzimidazole agents on glioblastoma cell viability *in vitro*. U87 (A), U87vIII (B), T98G (C), and U251 (D) GBM cells were incubated for 72 h with increasing concentrations of benzimidazole agents. Cell viability was assessed by Alamar Blue assay and expressed as percentage of untreated cells. Points, mean of at least four independent experiments; Bars, SEM.

To investigate the antiproliferative properties of benzimidazole agents *in vitro*, a range of human glioblastoma (GBM) cell lines was used (U87, U87vIII, T98G, and U251). As shown in Fig. 1, all tested compounds exerted dose‐dependent antiproliferative effects against all four GBM cell lines. Potency varied significantly between the different benzimidazoles, except in U251 cell line, which was highly sensitive to all four compounds. In all tested cell lines, the most active compound was mebendazole (MBZ) and the least potent was albendazole. MBZ displayed strong antiproliferative activity with IC_50_ values ranging from 288 ± 3 nm for U251 cell line to 2.1 ± 0.6 µm for the most resistant cell line, T98G.

### 3.2 *In silico* target prediction suggests multiple novel molecular targets for MBZ

**Fig. 2:**
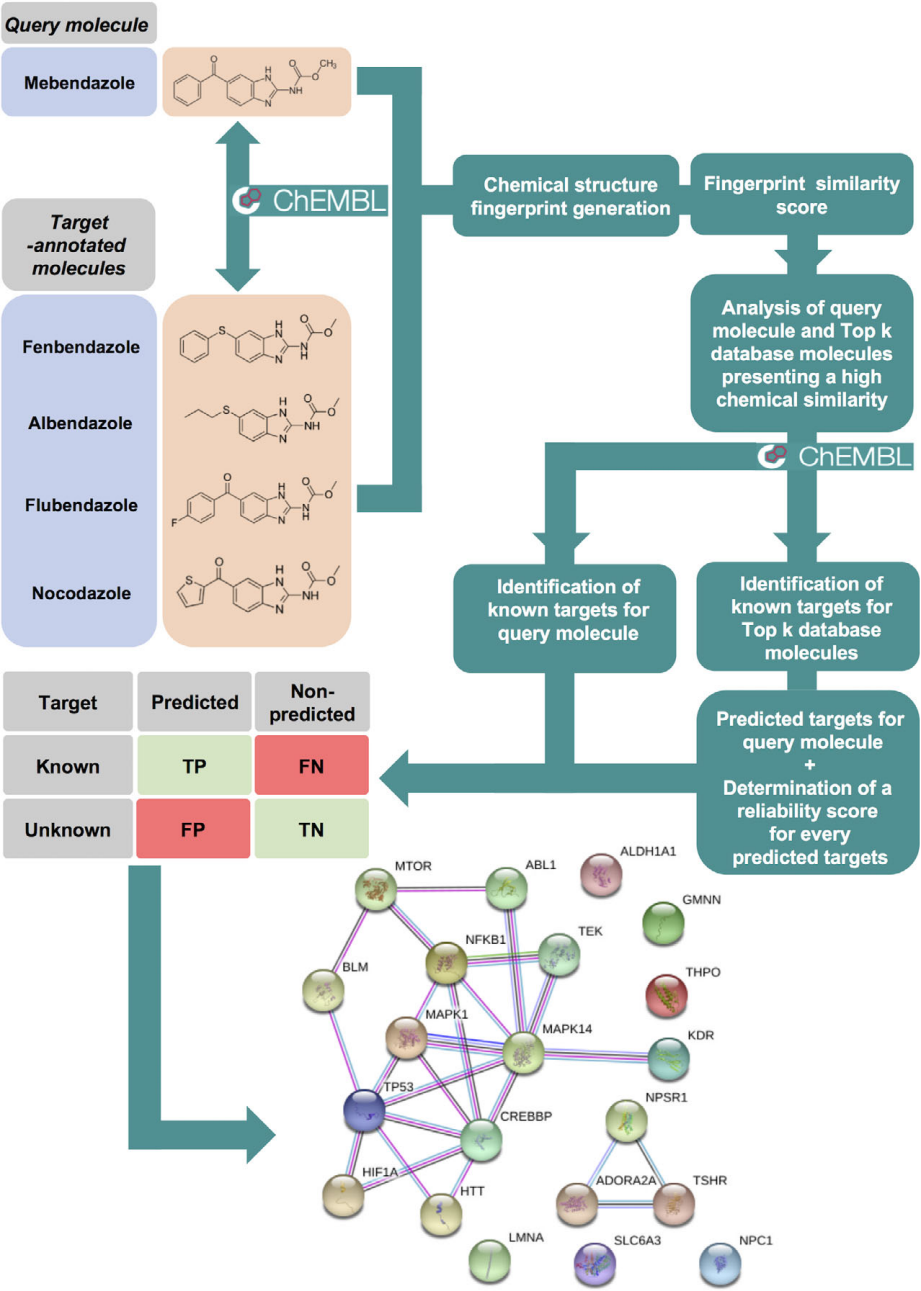
moltarpred ligand‐centric target prediction workflow. The most similar database molecules to the query molecule (mebendazole) are identified as previously detailed [27]. Known targets for these molecules are retrieved from the ChEMBL database. A reliability score for each query–target association prediction is calculated based on the proportion of the query's top hits binding to the predicted target. The protein–protein interaction network of the 21 human proteins predicted as targets of MBZ is provided (string‐db.org, version 11.0).

Computational methods are now being actively investigated as tools for identifying new therapeutic targets for repurposed drugs. Here, we used a data‐driven predictive tool called moltarpred [27]. Briefly, moltarpred (http://moltarpred.marseille.inserm.fr/) is a freely available web tool for predicting protein targets of small organic molecules. It is powered by a large knowledge base comprising 607 659 molecules and their known targets from the ChEMBL database [28]. moltarpred returns the most similar target‐annotated molecules to the user‐supplied query molecule, enabling the identification of a large amount of predictable targets based on those known in similar molecules. Using the current ChEMBL database, moltarpred can thus predict up to 4553 different proteins as putative molecular targets [4]. Here, we used this *in silico* tool to unveil novel putative molecular targets for MBZ (Fig. 2). We identified four other benzimidazole drugs, namely fenbendazole, flubendazole, albendazole, and nocodazole, among the 10 molecules most similar to MBZ (out of 607 659 target‐annotated molecules). MolTarPred predicted 21 human targets for this query molecule (Table 1). These include proteins involved in various biological processes, such as cell response to stress, signal transduction, and cell cycle regulation. Interestingly, some of these proteins are major established therapeutic targets in oncology (e.g., TP53, MTOR/FRAP1, HIF1‐α, VEGFR2/KDR, and ABL1).

**Table 1:** List of putative mebendazole targets identified by *in silico* target prediction.

Gene name	Full protein name	Main function
*ABL1*	Tyrosine protein kinase ABL	Kinase
*MAPK1*	MAP kinase ERK2	
*MAPK14*	MAP kinase p38 alpha	
*FRAP1*	Serine/threonine protein kinase mTOR	
*TEK*	Tyrosine protein kinase TIE‐2	
*KDR* ^a^	Vascular endothelial growth factor receptor 2	
*LGR3*	Thyroid‐stimulating hormone receptor	Receptor
*ADORA2A*	Adenosine A2a receptor	
*NPSR1*	Neuropeptide S receptor	
*NPC1*	Niemann–Pick C1 protein	Transporter
*SLC6A3*	Dopamine transporter	
*HIF1A*	Hypoxia‐inducible factor 1 alpha	Transcription factor
*NFKB1*	Nuclear factor (NF)‐kappa‐B p105 subunit	
*CREBBP*	CREB‐binding protein	Transcription co‐activator
*LMNA*	Prelamin A/C	Nuclear membrane
*GMNN*	Geminin	DNA replication inhibitor
*BLM*	Bloom syndrome protein	DNA helicase
*TP53*	Cellular tumor antigen p53	Tumor suppressor
*ALDH1A1*	Aldehyde dehydrogenase 1A1	Enzyme
*THPO*	Thrombopoietin	Hormone
*HTT*	Huntingtin	vesicular transport

^a^ Although KDR/VEGFR2 is a membrane receptor, its kinase activity is inhibited by benzimidazole agents [31].

### 3.3 MBZ inhibits ABL1, ERK2/MAPK1, and MAPK14/p38α *in vitro*, with particularly high potency against MAPK14/p38α

**Fig. 3:**
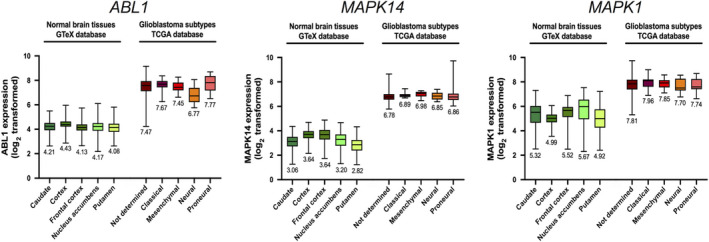
*ABL1*, *MAPK14*, and *MAPK1* gene expression in glioblastoma and normal brain tissue. Normal brain tissue and GBM tissue gene expression values were obtained from the GTeX and TCGA databases. Box plot representation showing relative Log2‐transformed gene expression for *ABL1* (left), *MAPK14* (middle), and *MAPK1* (right) in normal brain tissue [caudate (*n* = 246), cortex (*n* = 255), frontal cortex (*n* = 209), nucleus accumbens (*n* = 246), and putamen (*n* = 205)] and GBM subtypes [not determined (*n* = 455), classical (*n* = 17), mesenchymal (*n* = 27), neural (*n* = 17), and proneural (*n* = 24)] is shown. Average gene expression value is indicated below each box plot.

**Fig. 4:**
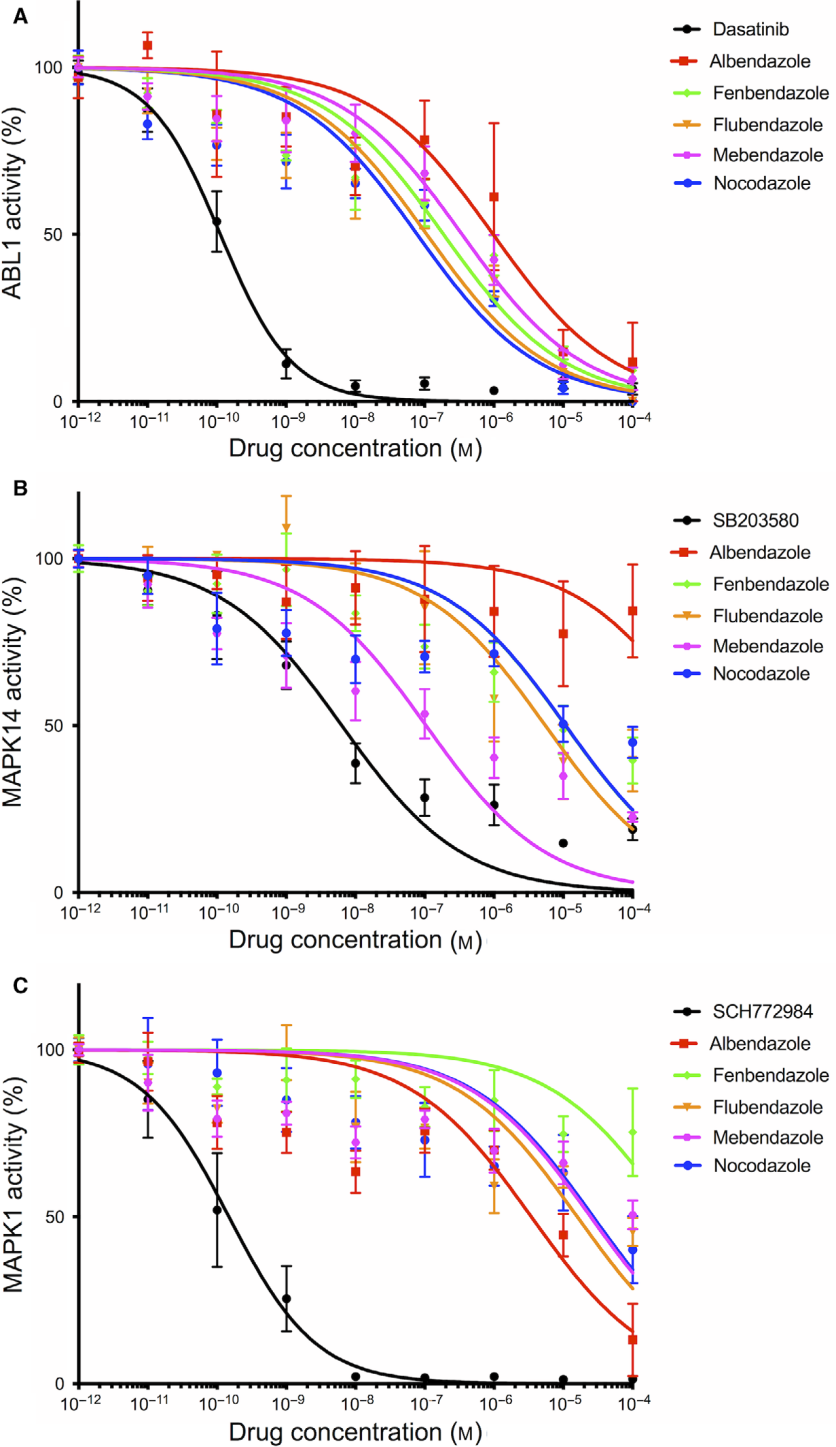
*In vitro* validation of protein kinase inhibition by benzimidazole agents. The concentration‐dependent inhibition of ABL1 (A), MAPK14 (B), and ERK2 (C) by benzimidazole agents was determined by kinase assay as described in Section 2. Kinase activities in the absence of inhibitor were set to 100%, and remaining activities at different drug concentrations are expressed relative to this value. Dasatinib, SB203580, and SCH772984 were used as positive control to inhibit the kinase activity of ABL1, MAPK14, and ERK2, respectively. Points, mean of at least four independent experiments; Bars, SEM.

Kinases have been among the most intensively pursued targets in oncology, leading to the approval of 46 kinase inhibitors for cancer treatment to date and over 150 kinase inhibitors currently in clinical trials [29, 30]. Interestingly, our transcriptomic analyses using freely available databases showed that 1/3 of the putative MBZ targets significantly upregulated in GBM as compared to normal brain tissue are kinases: ABL1, MAPK14/p38α, ERK2/MAPK1, and VEGFR2/KDR (Fig. 3 and Table S1). Since MBZ has been previously shown to inhibit the kinase activity of the latter *in vitro* [31], we focused our validation experiments on the other three kinases, whose expression was upregulated in GBM tissue. Indeed, their expression significantly increased from 4.23 ± 0.1, 3.34 ± 0.37, and 5.32 ± 0.43 to 7.45 ± 0.43, 6.84 ± 0.12, and 7.82 ± 0.22, respectively, for *ABL1*, *MAPK14*, and *MAPK1* gene expression in normal brain tissue compared to GBM tissue (Fig. 3 and Table S1). To experimentally validate the results of the *in silico* prediction, we performed functional assays to determine whether benzimidazoles were able to directly inhibit the activity of these kinases. We thus performed *in vitro* kinase assays on ABL1, MAPK14, and ERK2 kinases using a large range of concentrations (0.01 nm to 100 µm) of MBZ, albendazole, fenbendazole, flubendazole, and nocodazole. As illustrated in Fig. 4, benzimidazoles were able to inhibit the kinase activity of ABL1 and MAPK14 in a dose‐dependent manner and to a lesser extent for ERK2. All tested compounds showed differential potency against the three kinases, with nocodozale being the most potent at inhibiting ABL1 activity (IC_50_ = 78 ± 34 nm), MBZ at inhibiting MAPK14 activity (IC_50_ = 104 ± 46 nm), and albendazole at inhibiting ERK2 activity (IC_50_ = 3.4 ± 1.5 µm). The different IC_50_ values are summarized in Table 2. Collectively, these results confirm that MBZ inhibits all three tested predicted targets *in vitro*, with particularly high potency against MAPK14/p38α.

**Table 2:** IC_50_ values of benzimidazole agent in ABL1, ERK2, and MAPK14 *in vitro* kinase assays.

Drug	IC_50_ value (µm) ± SD		
	ABL1	ERK2	MAPK14
Albendazole	0.9 ± 0.6	3.4 ± 1.5	> 50
Fenbendazole	0.19 ± 0.07	> 50	10.7 ± 4.9
Flubendazole	0.11 ± 0.05	15.9 ± 8.0	5.5 ± 2.7
Mebendazole	0.35 ± 0.12	24.7 ± 10.4	0.10 ± 0.05
Nocodazole	0.08 ± 0.03	26.9 ± 16.2	2.8 ± 5.6

### 3.4 MBZ interacts directly with MAPK14 *in vitro* and inhibits its kinase activity *in cellulo*

**Fig. 5:**
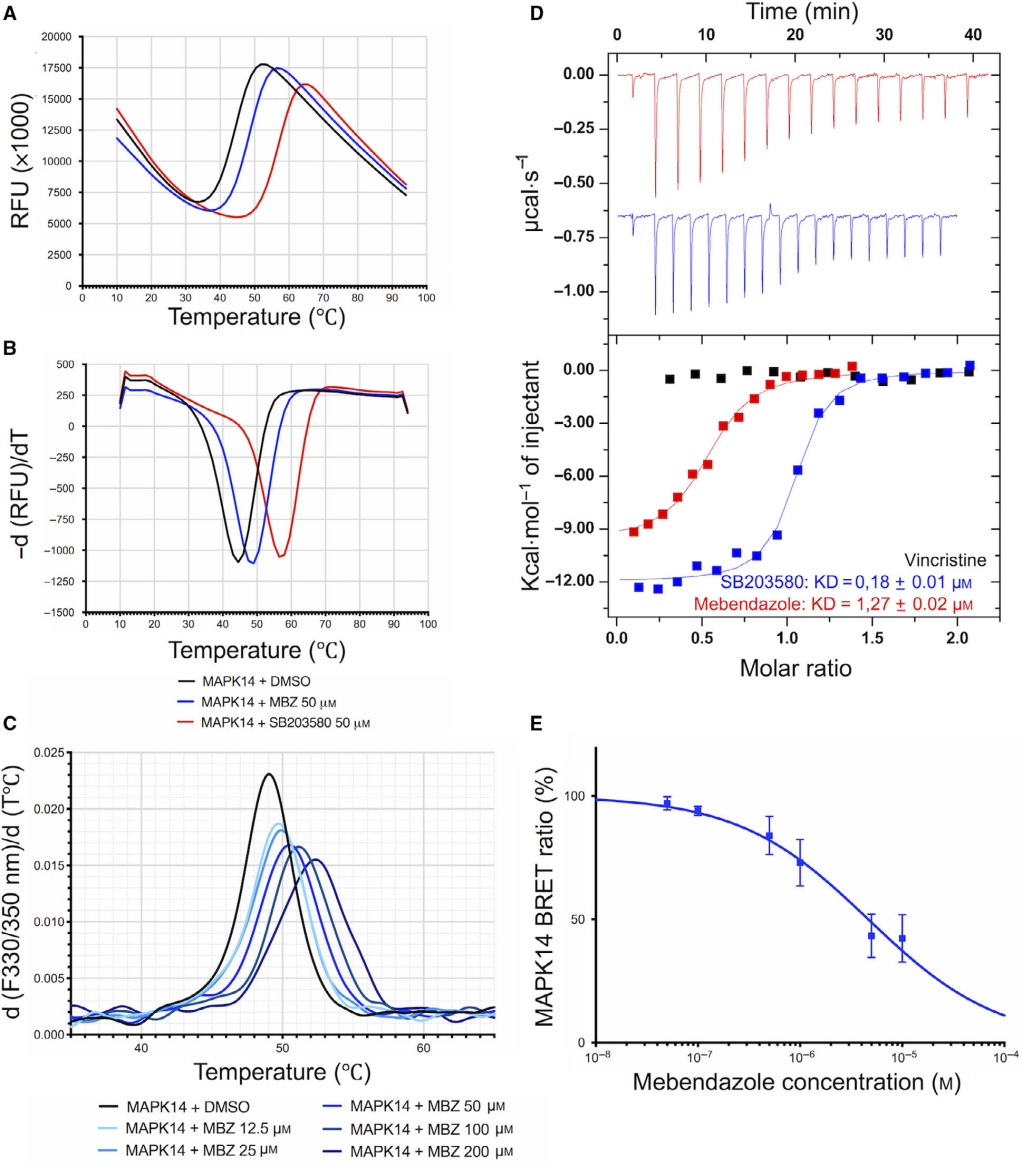
Biophysical characterization of MBZ binding to MAPK14 *in vitro* and *in cellulo* validation. Representative unfolding curves (A) and positive derivative [d(RFU)/d*T*] curves (B) of fluorescence‐based TSA performed on 5 µm of MAPK14 alone (black) or incubated with 50 µm of MBZ (blue) or SB203580 (red) over a temperature range of 10–95 °C. (C) First‐derivative curves of nanoDSF thermal shift on MAPK14 alone (5 µm) or in the presence of various concentrations of MBZ. (D) ITC measurements of the interaction between MBZ and MAPK14 protein using vincristine and SB203580 as negative and positive control, respectively. (E) NanoBRET target engagement assay with U87 cells transiently transfected with MAPK14‐NanoLuc fusion vector incubated with increasing concentrations of MBZ for 1 h. Points, mean of at least four independent experiments; Bars, SEM.

To characterize the interaction of MBZ with MAPK14 and perform orthogonal validation, we then employed a panel of established biophysical techniques, including TSA, nanoscale differential scanning fluorimetry (nanoDSF), and ITC. As represented in Fig. 5A,B, MAPK14 selective inhibitor, SB203580, was used as a positive control and induced an increase in MAPK14 thermostability at 50 µm (+12.7 °C). MBZ was also able to increase the thermostability of MAPK14, which shifted from 44.3 °C for the free form to 48 °C in the presence of MBZ at 50 µm (+3.7 °C). Similarly, MBZ was able to increase the thermostability of ABL1 (+6.3 °C) but to a lower extent than clinically approved ABL1 inhibitors, imatinib and dasatinib (+12.3 °C and +18.8 °C, respectively; Fig. S1). The direct binding of MBZ to MAPK14 was confirmed by nanoDSF, where increasing drug concentrations resulted in increased thermostability (Fig. 5C). Furthermore, MBZ binding to MAPK14 was validated by ITC measurements, which allowed us to quantify the dissociation constant of the MBZ‐MAPK14 complex (1.27 ± 0.02 µm; Fig. 5D). The thermodynamics parameters measured for MBZ exhibit a strong enthalpy component of −14 kcal·mol^−1^ compared to the −11.75 kcal·mol^−1^ measured for SB203580 (Fig. S2). Therefore, MBZ should engage the target with a favorable network of hydrogen bond interactions. SB203580 better *K*_d_ (0.18 ± 0.01 µm; Fig. 5D) is explained by a compensated favorable entropy component that could be partly due to the compound rigidity. Finally, in order to demonstrate that MBZ was able to inhibit MAPK14 activity in live GBM cells, we conducted NanoBRET™ target engagement intracellular kinase assay in U87 cells transfected with MAPK14‐NanoLuc^®^ fusion vector DNA. Here, we found that the BRET ratio decreased after MBZ treatment in a dose‐dependent manner (IC_50_ = 4.1 ± 1.1 µm). Taken together, these results show that MBZ is able to directly bind to MAPK14 and inhibit its kinase activity *in vitro* and *in cellulo*.

### 3.5 Prediction of the binding mode of MBZ within MAPK14 using molecular modeling

**Fig. 6:**
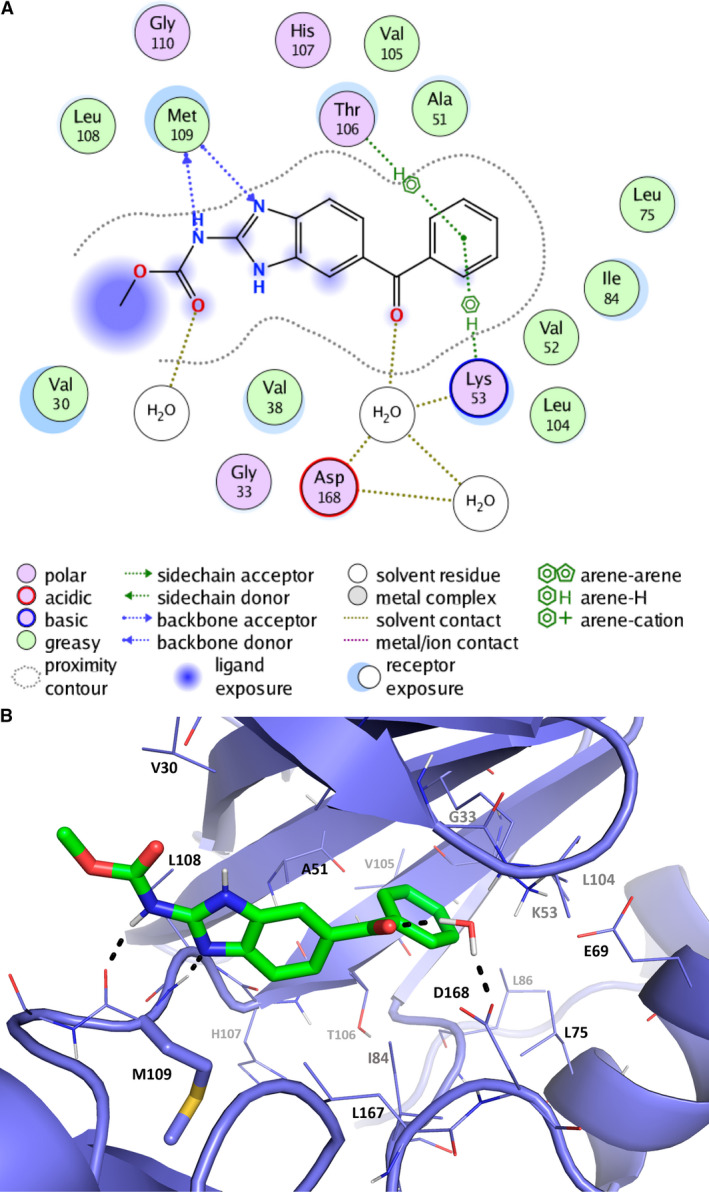
Proposed binding mode of mebendazole to MAPK14. The binding mode of MBZ to MAPK14 protein is depicted as 2D (A) and 3D diagrams (B), which were generated using the ‘Ligand interaction’ feature from moe and pymol software, respectively. The benzimidazole core is predicted to make hydrogen bond interaction with the hot spot M109, and the other aromatic moiety is deeply buried in a hydrophobic pocket of the binding site. MBZ and the side chain from M109 are displayed in sticks, and surrounding protein residues are depicted in lines. The backbone of the protein is shown as a cartoon representation, and key amino acids involved in MBZ binding are indicated.

More than 200 structures of MAPK14 (UniProt ID Q16539) are available from the Protein Data Bank (PDB) structural database. Kinases are known to be highly flexible proteins, and large structural rearrangements can be observed after the superimposition of all structures in the same referential. Docking simulations were performed on several PDB structures. The 3FLY one resulted in a coherent predicted binding mode for MBZ and its analog compounds. The postprocessed binding mode using seesar is displayed in Fig. 6 using both 3D and 2D imageries. As anticipated from the measured enthalpy data by ITC, several hydrogen bond interactions are predicted by the model. More precisely, the carbamate–benzimidazole moiety is expected to make two hydrogen bonds with backbone atoms from M109, a crucial hot spot of the ATP‐binding pocket. Besides, this aromatic core is also superimposed with the potent pyrido‐pyrimidin inhibitor from the 3FLY structure (IC_50_ = 4 nm). The carbonyl group between both aromatic rings of MBZ is also predicted, as the 3FLY ligand, to make a water‐mediated hydrogen bond with the side chain from D168 (Fig. S3A). This last feature was not included in the water‐free docking experiments but was highlighted by the seesar postprocessing where crystallographic waters from the reference PDB structure can be switched on/off. Finally, the terminal phenyl ring of MBZ is predicted to be superimposed with the difluorophenyl moiety from the 3FLY pyrido‐pyrimidin inhibitor, deeply buried within a small and well‐defined hydrophobic pocket delimited by V38, A51, K53, L75, L86, and L104 residues. The molecular modeling study was also able to explain the lower affinity of albendazole for MAPK14 from its predicted binding mode. The single difference between albendazole and MBZ ligands is their terminal end: a rigid aromatic ring for MBZ and a flexible aliphatic chain for albendazole. Therefore, albendazole is predicted to make less Van der Waals interactions between its hydrophobic side chain within the hydrophobic pocket (Fig. S3B). Besides, albendazole lacks the carbonyl spacer that is predicted to be involved in water‐mediated hydrogen bonds with the protein (K53 and D168). Finally, this ligand also exhibits more flexibility that is expected to decrease the affinity from an entropic point of view.

Kinase assays also revealed that MBZ displays high affinity for ABL1 (IC_50_ = 350 nm) and almost no affinity for MAPK1/ERK2 (IC_50_ = 25 µm). This activity cliff was also investigated using molecular modeling studies. In agreement with the kinase assay, the proposed MBZ‐binding mode to ABL1 exhibits a highly similar interaction pattern with MAPK14. The loss of a water‐mediated hydrogen bond interaction with ABL1 (the aspartic acid 168 in MAPK14 is replaced by a phenylalanine in ABL1) could explain the slightly lower affinity observed for MBZ (Fig. S3B,C). The equivalent analysis conducted on MAPK1 identified severe clashes between the terminal aromatic ring of MBZ and an asparagine from the protein. The bulkier hydrophilic side chain of N103 in MAPK1 replaces a threonine in MAPK14 (T106) and in ABL‐1 (T315). The resulting reduced cavity clearly explains the observed activity cliff for MAPK1 (Fig. S3D).

### 3.6 MAPK14 is a therapeutic target in GBM

**Fig. 7:**
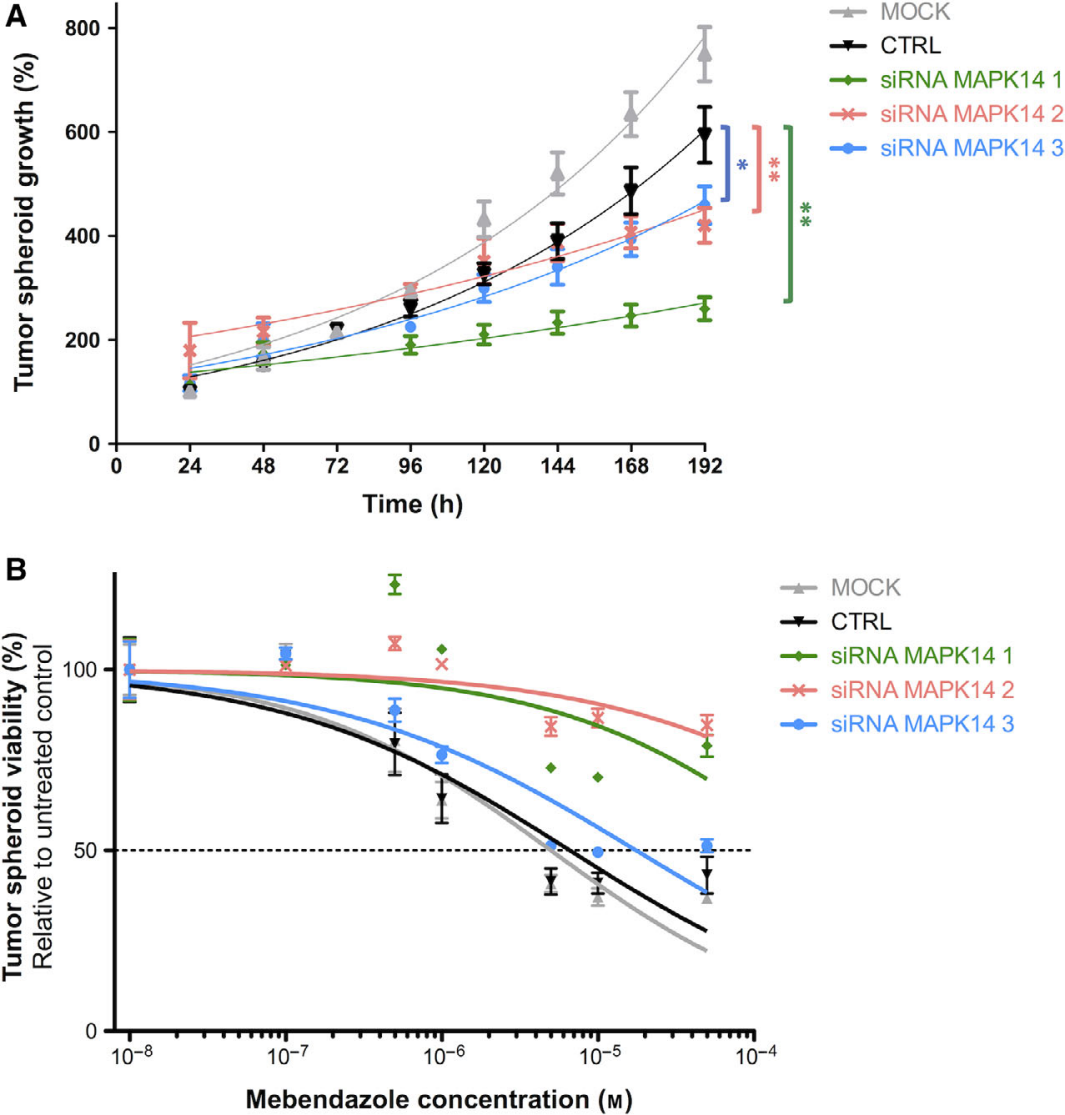
Functional validation of MAPK14 as a key molecular target of mebendazole in glioblastoma cells. (A) Growth curves of 3D tumor spheroids formed by dsRed‐expressing U87 cells either untransfected (mock) or transfected with negative control or MAPK14‐targeting siRNA. Spheroid growth was assessed by daily fluorescence measurements at 575/620 nm. (B) Dose–response curve of dsRed‐expressing U87 cells, either untransfected (mock) or transfected with negative control or MAPK14‐targeting siRNA, incubated with increasing concentrations of MBZ for 192 h. Points, mean of at least four independent experiments; Bars, SEM.

The cytotoxic activity of benzimidazoles significantly correlated with their ability to inhibit MAPK14 kinase activity *in vitro* in 2 out of 4 tested GBM cell lines, and a similar trend was observed in the other two cell lines (Table S2). Interestingly, this was not observed with ABL1 and ERK2 kinase activity. We therefore performed *MAPK14* gene silencing in GBM cells to confirm its role as a key molecular target of MBZ. *MAPK14* gene expression was knocked down in dsRed‐expressing U87 cells by transfection with three different siRNA sequences (Fig. S4). This 75–90% knockdown of *MAPK14* expression resulted in a significant decrease in tumor spheroid growth *in vitro* (Fig. 7A). While negative control siRNA‐transfected spheroids grew by 595% in 7 days, they only grew by 260–460% when transfected with MAPK14‐targeting siRNA (*P* < 0.05). Similarly, spheroid doubling time increased from 75 h following transfection with negative control siRNA to 172, 149, and 100 h following transfection with MAPK14 siRNA sequences #1, #2, and #3, respectively. Consistent with the role of MAPK14 as a critical molecular target of MBZ, *MAPK14* gene silencing significantly decreased the sensitivity of GBM cells to the drug (Fig. 7B). The IC_50_ value of MBZ after 192‐h incubation increased from 5 and 6.6 µm in mock and control siRNA‐transfected cells, respectively, to 17.6 µm in cells transfected with MAPK14‐targeting siRNA sequence #3 and > 50 µm in cells transfected with the other two siRNA sequences. These results confirmed the role of MAPK14 in GBM cell response to MBZ.

## 4 Discussion

Drug repurposing, which consists in using already‐approved drugs in new medical indications, has become an attractive therapeutic strategy in oncology. There are, however, two major hurdles to overcome for successful drug repurposing strategies: (a) identifying the right drug for the right situation (i.e., disease type, patient population, and clinical setting) and (b) deciphering the new mechanism(s) of action of repurposed drugs in the context of cancer. In this study, we established an innovative framework to rapidly identify and validate the molecular target(s) of MBZ, an antihelminthic agent, currently repurposed for the treatment of GBM. Our results revealed for the first time a crucial role of MAPK14 in its mechanism of action.

The conventional approaches to drug development in oncology take on average 12–15 years and the median cost of anticancer drugs at the time of approval went from less than $100 per month in the 1990s to over $10 000 per month currently, threatening national healthcare systems worldwide [5, 32]. In this context, drug repurposing holds great promise to develop safe, effective, and inexpensive therapeutic options that can be made readily available to cancer patients regardless of their socioeconomic status [5, 33]. Over the last decade, MBZ has been recognized as an attractive candidate for drug repurposing in various models of human cancers [7, 8, 9, 10, 11, 12]. Its anticancer properties were notably demonstrated in gliomas [11, 14, 34], and there are currently three ongoing clinical trials in high‐grade gliomas in both adult and pediatric patients (NCT01729260, NCT02644291, and NCT01837862). Furthermore, a recent phase I trial demonstrated that administration of MBZ concomitantly with radiotherapy and temozolomide chemotherapy was safe in high‐grade glioma patients [35].

Although multiple studies have highlighted different mechanisms of action of MBZ in cancer cells [12, 13, 14, 15, 16], including its effects on the microtubule network [8, 11, 14, 17, 18], its precise molecular target(s) in tumor cells remained to be ascertained. Herein, we used *in silico* target prediction to unveil novel therapeutic targets for MBZ in GBM. We foresaw 21 putative targets, of which four had been previously shown to be modulated by benzimidazole agents *in vitro*: ABL1, VEGFR2, mTOR, and aldehyde dehydrogenase [19, 31, 36, 37]. Our methodology thus identified 17 novel putative targets of MBZ. We also observed that 12 of the 21 predicted targets are significantly overexpressed in GBM as compared to normal brain tissue, which may explain why MBZ is particularly effective against brain tumors [11, 13, 14, 17, 38].

We focused our validation experiments on three major kinases involved in cancer: the tyrosine kinase ABL1, the mitogen‐activated protein kinase (MAPK) member ERK2, and one of the four p38 MAPKs, MAPK14. All of these proteins have already been shown to play a key role in the pathophysiology and drug resistance of GBM. Indeed, while ABL1 was found to be involved in DNA repair upon irradiation, thus contributing to radioresistance in GBM cells [39], a crucial role in linking RNA processing with signal transduction of ERK2 has recently been unveiled [40]. Moreover, activation of the MAPK14 signaling pathway has been previously shown to correlate with poor prognosis in GBM patients, increased tumor invasiveness, and aggressive phenotype [41, 42, 43, 44, 45]. Here, *in vitro* kinase assays confirmed that MBZ could directly inhibit the kinase activity of these proteins, thus confirming their status of direct molecular targets of MBZ. Moreover, we found that all three kinases are overexpressed in GBM as compared to normal brain, thus providing an opportunity for targeted strategy in this deadly form of brain tumor.

MAPK14 is one of the four p38 MAPKs that play an important role in the cascades of cellular responses evoked by extracellular stimuli such as proinflammatory cytokines or physical stress leading to direct activation of transcription factors [46]. For many years, p38 MAPK kinases have been considered as attractive targets for chronic inflammatory disease therapy. p38 MAPK inhibitors have taken a leap forward through the development of many compounds for different pathologies, including cancers [46, 47]. Here, we have discovered that MBZ is particularly potent at inhibiting MAPK14 kinase activity *in vitro*. Molecular modeling studies also revealed that the carbamate‐benzimidazole moiety of MBZ engages with the critical M109 hot spot of the catalytic site of MAPK14. Interestingly, it has been demonstrated that the binding mode of selective MAPK14 inhibitors is characterized by a compound‐induced peptide flip between M109 and G110 [48]. Besides, some MBZ–urea inhibitors are known to bind the hinge region from other kinases such as VEGFR2 [49], and one reference structure is publicly available from the PDB database (PDB ID 2OH4). Interestingly, the carbamate‐benzimidazole moiety adopts the same interaction pattern with the homologous hot spot from VEGFR2 protein. These structural data strengthen the reliability of the predicted binding mode of the MBZ series within the MAPK14‐binding site. Furthermore, additional molecular modeling studies supported MBZ selectivity profile, that is, a similar potency between MAPK14 and ABL1 but almost no activity for MAPK1.

The MAPK14/p38α signaling pathway has been classically considered a tumor suppressor. However, several studies have also demonstrated the protumorigenic activities of MAPK14, which facilitates the survival and proliferation of tumor cells [46]. In our study, the use of RNA interference revealed that MAPK14 expression is crucial to GBM tumor growth in a spheroid model, confirming the therapeutic potential of targeting MAPK14 in GBM. Consistently, MAPK14 selective inhibitor, LY2228820 (ralimetinib), has been shown to produce significant tumor growth delay in multiple cancer models, including GBM [50, 51], and has now entered clinical trials [52]. Moreover, recent evidence suggests that MAPK14 is also involved in drug resistance. For instance, response to cisplatin can be enhanced by MAPK14 inhibition, resulting in ROS‐dependent upregulation of the JNK pathway in colon and breast cancer cells [53]. Similarly, MAPK14 confers resistance to irinotecan in TP53‐defective colon cancer cells by inducing prosurvival autophagy [54]. In line with this, MAPK14 was found to play a critical role in regulating response to temozolomide treatment in GBM [55, 56]. These studies along with the results presented herein strongly suggest that targeting MAPK14 with MBZ or other pharmacological inhibitors represents a promising strategy to enhance chemotherapy efficacy in cancer, including temozolomide efficacy against GBM.

## 5 Conclusions

In conclusion, we have found consistent evidence that further supports the use of MBZ as a promising repurposed drug in various tumor types and especially in brain cancers. We have demonstrated for the first time that MBZ is a potent inhibitor of MAPK14, which would directly contribute to its anticancer properties in GBM. Our results could thus open new therapeutic avenues for the development of MAPK14 inhibitor combination therapies in GBM and other human diseases. More broadly, we have established a framework for the rapid identification and functional validation of novel molecular targets that could be applied to other repurposed drugs, and therefore enable the development of innovative therapeutic strategies for unmet medical needs.

## Conflict of interest

The authors declare no conflict of interest.

## Author contributions

EP conceived the study, analyzed the data, and wrote and revised the manuscript. JA‐B performed most experiments, analyzed the data, and drafted parts of the manuscript. KC purified the MAPK14 protein and performed TSA and ITC experiments with SB. MLG undertook the transcriptomic analyses, while LH and SB performed the molecular modeling work. All three wrote and revised parts of the manuscript. MF performed the TSA experiments on ABL1 and PT and FD the nanoDSF experiments. YC and XM contributed to data analysis and manuscript preparation. PB performed the *in silico* target prediction. All authors read the manuscript and made comments in order to improve it.

## Supporting information


**Fig. S1.** Biophysical characterization of MBZ binding to ABL1 *in vitro*. Representative unfolding curves (A) and positive derivative [d(RFU)/dT] curves (B) of fluorescence‐based TSA performed with 4 µM of the kinase domain of ABL1 alone (*green*) or incubated with 200 µM of MBZ (*grey*), imatinib (*red*) or dasatinib (*blue*) over a temperature range of 10‐95 °C. *RFU*, relative fluorescence unit.
**Fig. S2.** Thermodynamics parameters of MBZ binding to MAPK14. (A) Histogram of the thermodynamics parameters measured by ITC for SB203580 (*black*) and MBZ (*grey*) binding to MAPK14. (B) Summary table of the thermodynamics parameters (mean of at least 3 independent experiments).
**Fig. S3.** Binding mode comparisons of MBZ with 3FLY ligand and albendazole. (A) Comparison of the binding mode of MBZ (*green*) with the pyrido‐pyrimidin inhibitor (*yellow*) from the 3FLY crystal structure. (B) Comparison of the binding mode of MBZ (*green*) and albendazole (*pink*). (C) Docking model of MBZ (*green*) in ABL‐1 (*black*) generated from the PDB ID: 1M52. (D) Alignment and comparison of MAPK14 (*blue*), ABL‐1 (*black*) and MAPK1 (*orange*) (PDB ID: 4ZZN) exhibiting the bulkier N103 amino acid in MAPK1 responsible for the loss of MBZ activity. Ligands and surrounding protein sidechains are displayed as sticks and lines, respectively. The backbone from the protein is shown as cartoon representation.
**Fig. S4.** *MAPK14* gene silencing by RNA interference. Histogram of *MAPK14* gene expression, relative to housekeeping gene *YWHAZ*, in DsRed‐expressing U87 cells following 48h transfection with negative ctrl (CTRL; *black*) and 3 different MAPK14 siRNA sequences (untransfected, MOCK; *grey*). Mean of 3 independent experiments +/‐ s.d; ***, p < 0.001.
**Table S1.** Average gene expression of MBZ putative targets in glioblastoma and normal brain tissue.
**Table S2.** Pearson correlation between the cytotoxic activity of benzimidazole agents and their inhibitory effects on ABL1, ERK2 and MAPK14 in 4 GBM cell lines.
