# Supplementary material for: In silico molecular target prediction unveils mebendazole as a potent MAPK14 inhibitor
Source: Mol Oncol. 2020 Oct 18;14(12):3083–99. doi: 10.1002/1878-0261.12810 (PMC7718943; doi:10.1002/1878-0261.12810)
Supplement: Supplementary file 1 — Fig. S1. Biophysical characterization of MBZ binding to ABL1 in vitro. Representative unfolding curves (A) and positive derivative [d(RFU)/dT] curves (B) of fluorescence‐based TSA performed with 4 µM of the kinase domain of ABL1 alone (green) or incubated with 200 µM of MBZ (grey), imatinib (red) or dasatinib (blue) over a temperature range of 10‐95 °C. RFU, relative fluorescence unit. Fig. S2. Thermodynamics parameters of MBZ binding to MAPK14. (A) Histogram of the thermodynamics parameters measured by ITC for SB203580 (black) and MBZ (grey) binding to MAPK14. (B) Summary table of the thermodynamics parameters (mean of at least 3 independent experiments). Fig. S3. Binding mode comparisons of MBZ with 3FLY ligand and albendazole. (A) Comparison of the binding mode of MBZ (green) with the pyrido‐pyrimidin inhibitor (yellow) from the 3FLY crystal structure. (B) Comparison of the binding mode of MBZ (green) and albendazole (pink). (C) Docking model of MBZ (green) in ABL‐1 (black) generated from the PDB ID: 1M52. (D) Alignment and comparison of MAPK14 (blue), ABL‐1 (black) and MAPK1 (orange) (PDB ID: 4ZZN) exhibiting the bulkier N103 amino acid in MAPK1 responsible for the loss of MBZ activity. Ligands and surrounding protein sidechains are displayed as sticks and lines, respectively. The backbone from the protein is shown as cartoon representation. Fig. S4. MAPK14 gene silencing by RNA interference. Histogram of MAPK14 gene expression, relative to housekeeping gene YWHAZ, in DsRed‐expressing U87 cells following 48h transfection with negative ctrl (CTRL; black) and 3 different MAPK14 siRNA sequences (untransfected, MOCK; grey). Mean of 3 independent experiments +/‐ s.d; ***, p < 0.001. Table S1. Average gene expression of MBZ putative targets in glioblastoma and normal brain tissue. Table S2. Pearson correlation between the cytotoxic activity of benzimidazole agents and their inhibitory effects on ABL1, ERK2 and MAPK14 in 4 GBM cell lines. [file MOL2-14-3083-s001.docx]

**Supplementary Table 1.** Average gene expression of MBZ putative targets in glioblastoma and normal brain tissue.

| **Cell line** | **Pearson correlation (R^2^ ; p value)** | | |
| --- | --- | --- | --- |
|  | **ABL1** | **ERK2** | **MAPK14** |
| **U87** | **0,488**  p=0.30 | **0,009**  p=0.91 | **0,746**  p=0.14 |
| **U87vIII** | **0,810**  p=0.10 | **0,358**  p=0.40 | **0,996**  p=0.002** |
| **T98G** | **0,874**  p=0.065 | **0,518**  p=0,28 | **0,966**  p=0.017* |
| **U251** | **0,611**  p=0.22 | **0,032**  p=0.82 | **0,821**  p=0.094 |

**Supplementary Table 2.** Pearson correlation between the cytotoxic activity of benzimidazole agents and their inhibitory effects on ABL1, ERK2 and MAPK14 in 4 GBM cell lines.


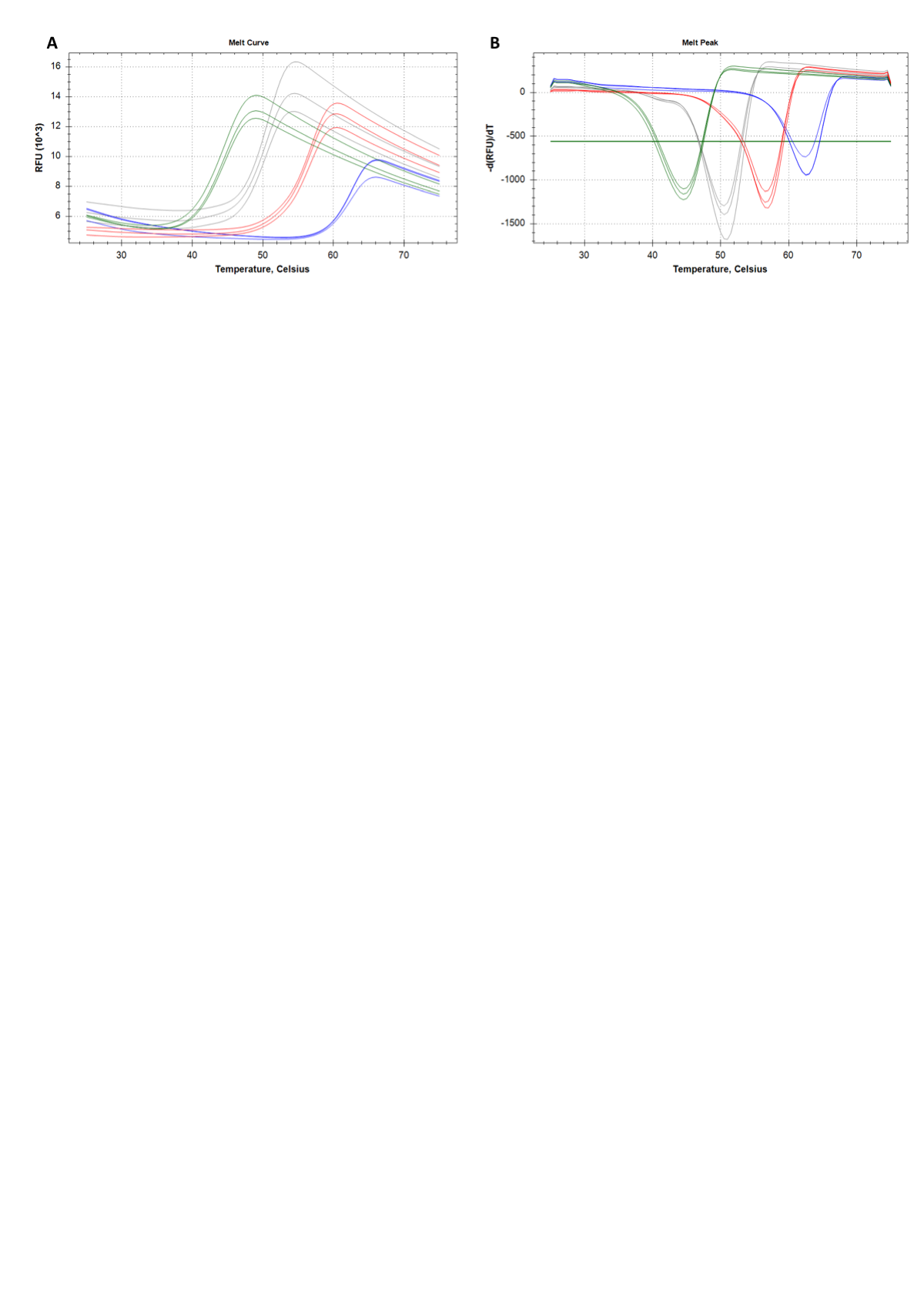


**Supplementary Figure 1. Biophysical characterization of MBZ binding to ABL1 *in vitro.*** Representative unfolding curves (**A**) and positive derivative [d(RFU)/dT] curves (**B**) of fluorescence-based TSA performed with 4 µM of the kinase domain of ABL1 alone (*green*) or incubated with 200 µM of MBZ (*grey*), imatinib (*red*) or dasatinib (*blue*) over a temperature range of 10-95°C. *RFU*, relative fluorescence unit.


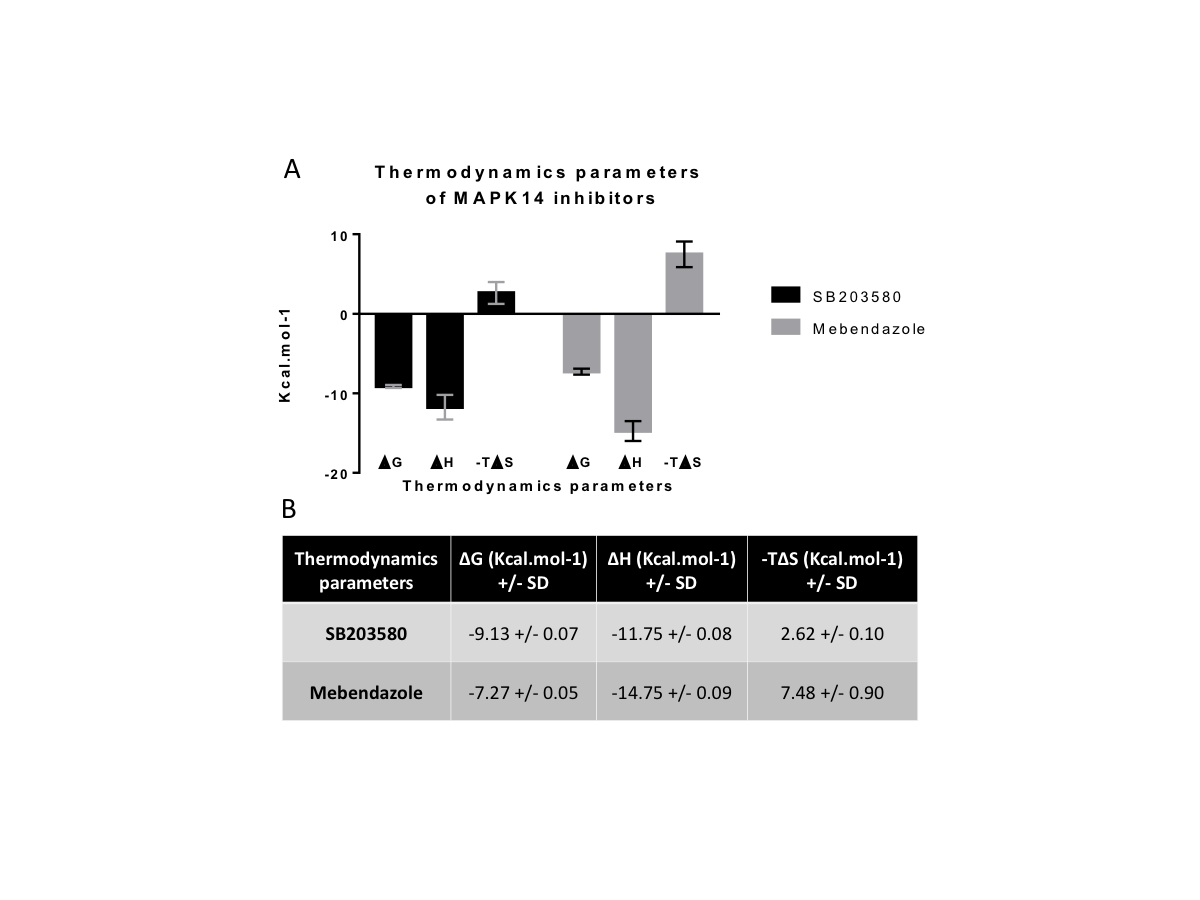


**Supplementary Figure 2. Thermodynamics parameters of MBZ binding to MAPK14*.*** (**A**) Histogram of the thermodynamics parameters measured by ITC for SB203580 (*black*) and MBZ (*grey*) binding to MAPK14. (**B**) Summary table of the thermodynamics parameters (mean of at least 3 independent experiments).


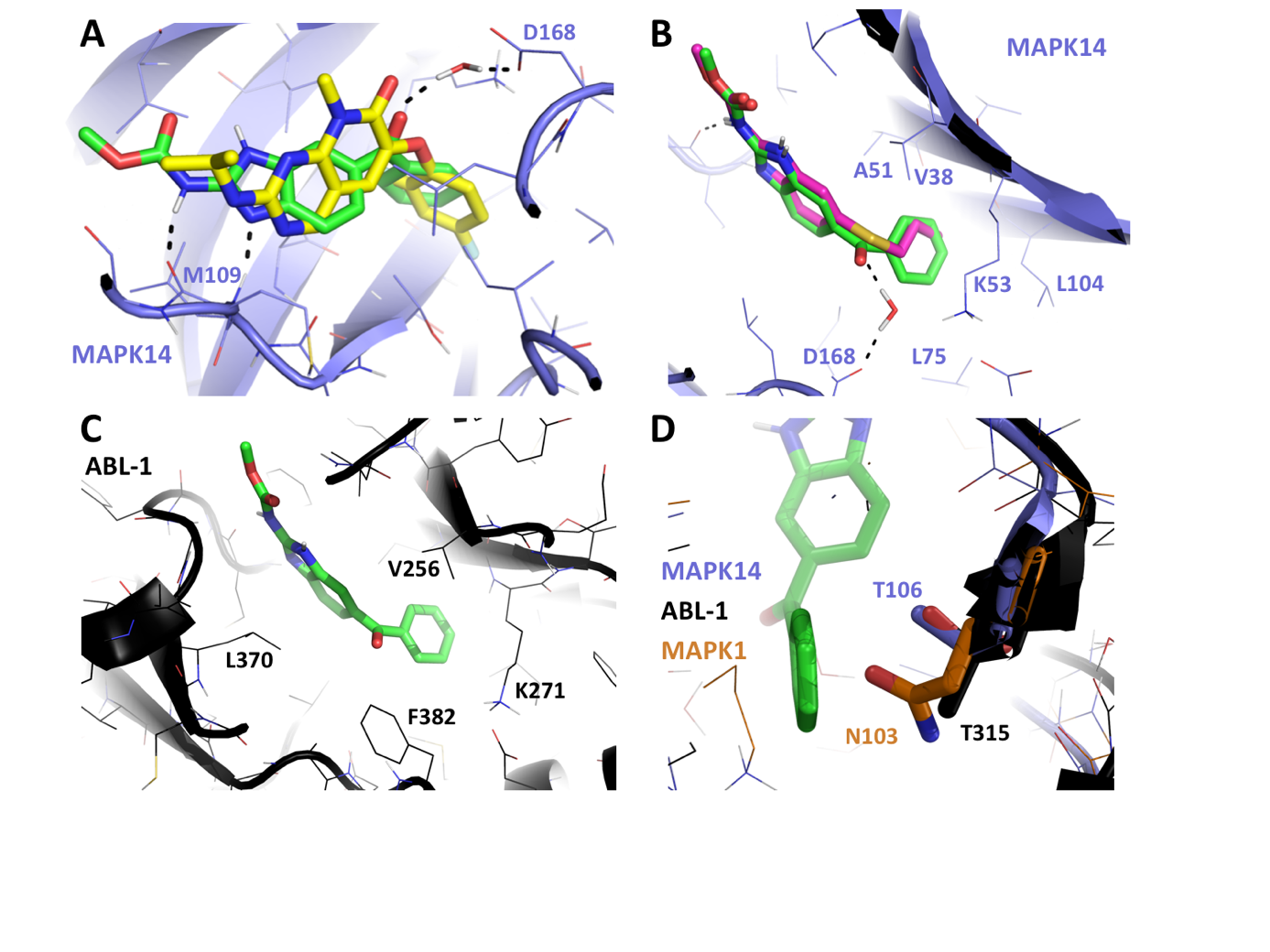


**Supplementary Figure 3. Binding mode comparisons of MBZ with 3FLY ligand and albendazole.** (**A**) Comparison of the binding mode of MBZ (*green*) with the pyrido-pyrimidin inhibitor (*yellow*) from the 3FLY crystal structure. (**B**) Comparison of the binding mode of MBZ (*green*) and albendazole (*pink*). (**C**) Docking model of MBZ (*green*) in ABL-1 (*black*) generated from the PDB ID: 1M52. (**D**) Alignment and comparison of MAPK14 (*blue*), ABL-1 (*black*) and MAPK1 (*orange*) (PDB ID: 4ZZN) exhibiting the bulkier N103 amino acid in MAPK1 responsible for the loss of MBZ activity. Ligands and surrounding protein sidechains are displayed as sticks and lines, respectively. The backbone from the protein is shown as cartoon representation.


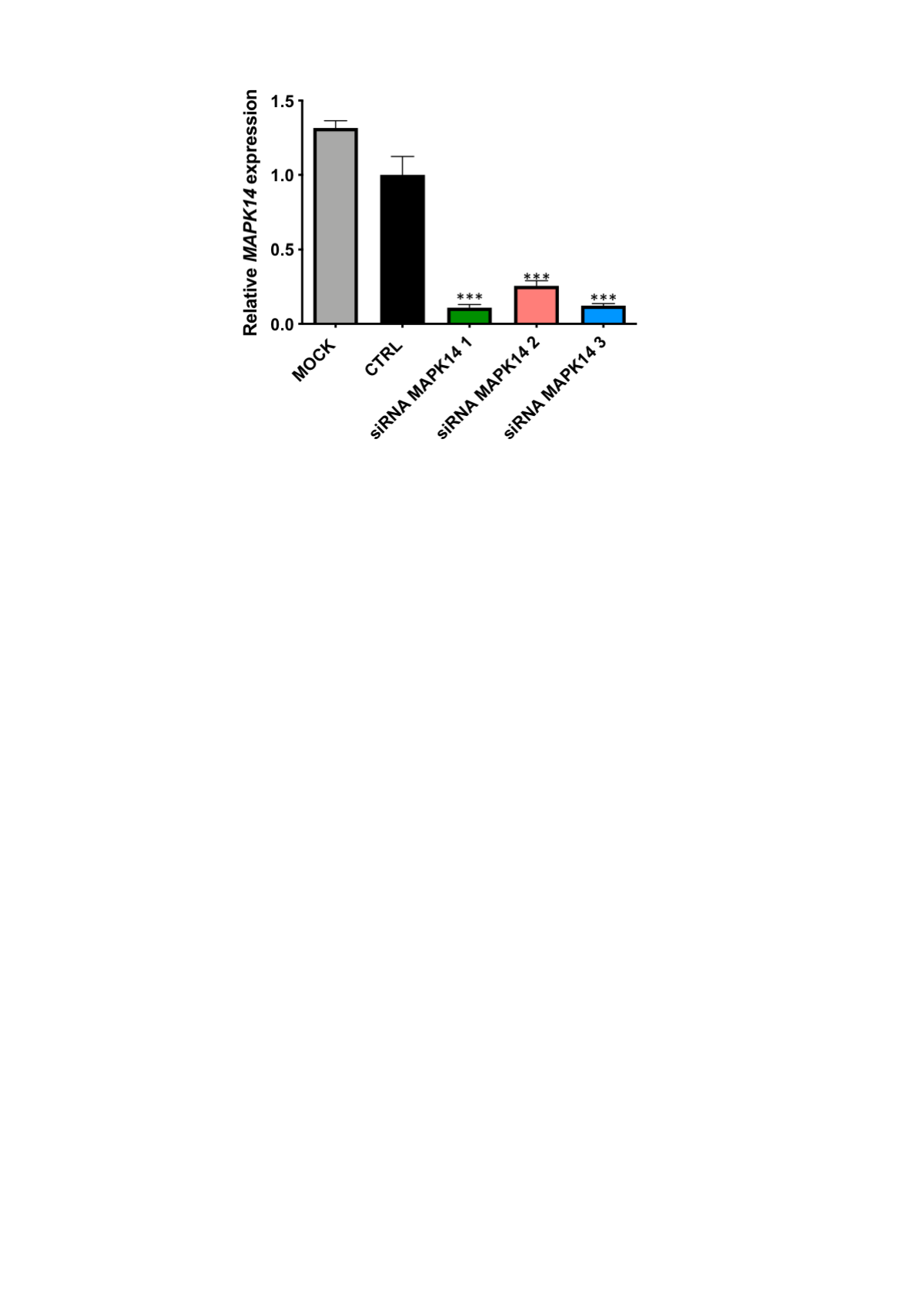


**Supplementary Figure 4. *MAPK14* gene silencing by RNA interference.** Histogram of *MAPK14* gene expression, relative to housekeeping gene *YWHAZ*, in DsRed-expressing U87 cells following 48h transfection with negative ctrl (CTRL; *black*) and 3 different MAPK14 siRNA sequences (untransfected, MOCK; *grey*). Mean of 3 independent experiments +/- s.d; ***, p<0.001.
